# Hippocampal Remapping and Its Entorhinal Origin

**DOI:** 10.3389/fnbeh.2017.00253

**Published:** 2018-01-04

**Authors:** Patrick Latuske, Olga Kornienko, Laura Kohler, Kevin Allen

**Affiliations:** Department of Clinical Neurobiology, German Cancer Research Center (DKFZ), Medical Faculty of Heidelberg University, Heidelberg University, Heidelberg, Germany

**Keywords:** place cells, entorhinal cortex, grid cell, remapping, navigation, hippocampus, memory

## Abstract

The activity of hippocampal cell ensembles is an accurate predictor of the position of an animal in its surrounding space. One key property of hippocampal cell ensembles is their ability to change in response to alterations in the surrounding environment, a phenomenon called remapping. In this review article, we present evidence for the distinct types of hippocampal remapping. The progressive divergence over time of cell ensembles active in different environments and the transition dynamics between pre-established maps are discussed. Finally, we review recent work demonstrating that hippocampal remapping can be triggered by neurons located in the entorhinal cortex.

## 1. Hippocampal neurons and mapping

In the early seventies, John O'Keefe started to investigate the firing properties of neurons in the hippocampus of awake rats (O'Keefe and Dostrovsky, 1971). At the time, most scientists interested in the role of the hippocampus focused on characterizing the effect of hippocampal lesions on different memory tasks (Douglas, 1967; Olton et al., 1979; Squire, 1992). O'Keefe asked a different question: what type of information is encoded in the firing activity of hippocampal neurons? He reported on a small subset of 8 hippocampal neurons that “responded solely or maximally when the rat was situated in a particular part of the testing platform facing in a particular direction.” He proposed that “the hippocampus provides the rest of the brain with a spatial reference map” (O'Keefe and Dostrovsky, 1971; O'Keefe and Nadel, 1978).

These early observations have been refined and extended, and it is now clear that the current location of an animal is one of the most prominent variables encoded in the activity of hippocampal excitatory neurons. Individual hippocampal neurons, called *place cells*, typically have one or two regions of high firing activity which are referred to as *place fields*. Firing fields can be located anywhere in an environment and their position usually remains stable in time if no changes are made to the recording environment (Muller et al., 1987).

**Figure 1:**
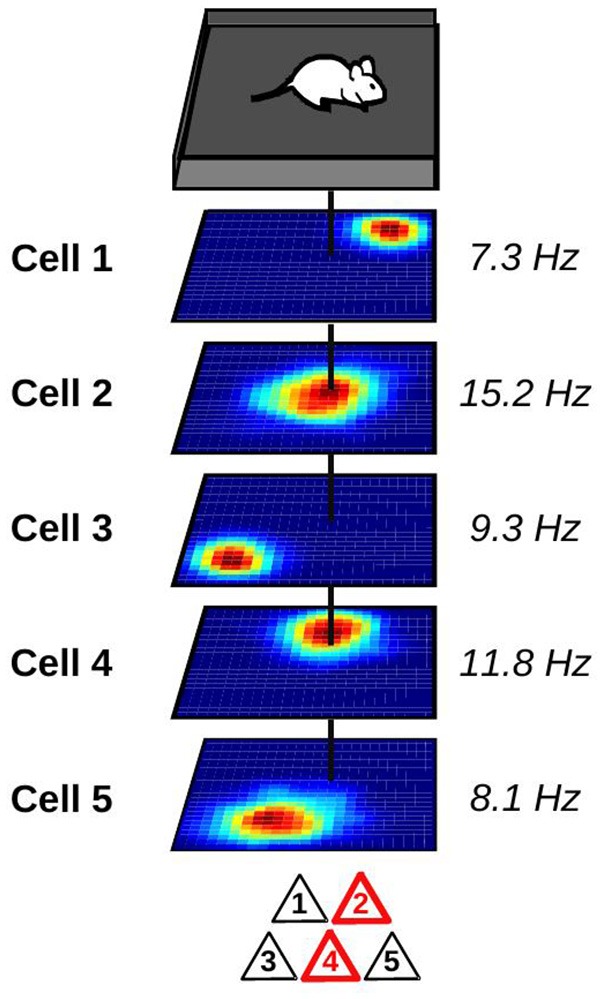
Representation of a hippocampal place cell ensemble. Top: mouse foraging in a square enclosure. Middle: stack of spatial firing rate maps of five hippocampal pyramidal cells (dark blue: silent; red: maximum firing rate). Numbers to the right indicate the peak firing rate of each cell. Vertical black line indicates the current location of the animal. Bottom: at the current location of the animal, only a subset of place cells is co-active and forms a cell ensemble. These active neurons are depicted in red.

With the development of new recording techniques, it has become possible to track the activity of large groups of hippocampal neurons in awake animals (Wilson and McNaughton, 1993; Pfeiffer and Foster, 2013; Ziv et al., 2013). As expected, place cells with overlapping firing fields tend to fire together. Neurons with this temporally and spatially correlated activity act as a functional unit called *cell ensemble* which encodes the current position of the animal (Figure 1). By monitoring the activity of cell ensembles, one can accurately predict the current location of an animal (Wilson and McNaughton, 1993; Zhang et al., 1998; Jensen and Lisman, 2000; Harris et al., 2003; van de Ven et al., 2016). The set of cell ensembles active in one environment forms an internal map of the environment.

But what happens to hippocampal cell ensembles when an animal moves to a different environment or when alterations are made to its surrounding? Often, these external changes cause a reorganization of the cell ensembles representing the position of the animal. This phenomenon is referred to as *remapping* and will be the subject of this review. In a first section, we cover experiments that characterize the different types of hippocampal remapping. In a second section, we review recent work indicating that one trigger of hippocampal remapping is located in the entorhinal cortex.

## 2. Hippocampal remapping

### 2.1. Global remapping: orthogonal reorganization of cell ensembles

**Figure 2:**
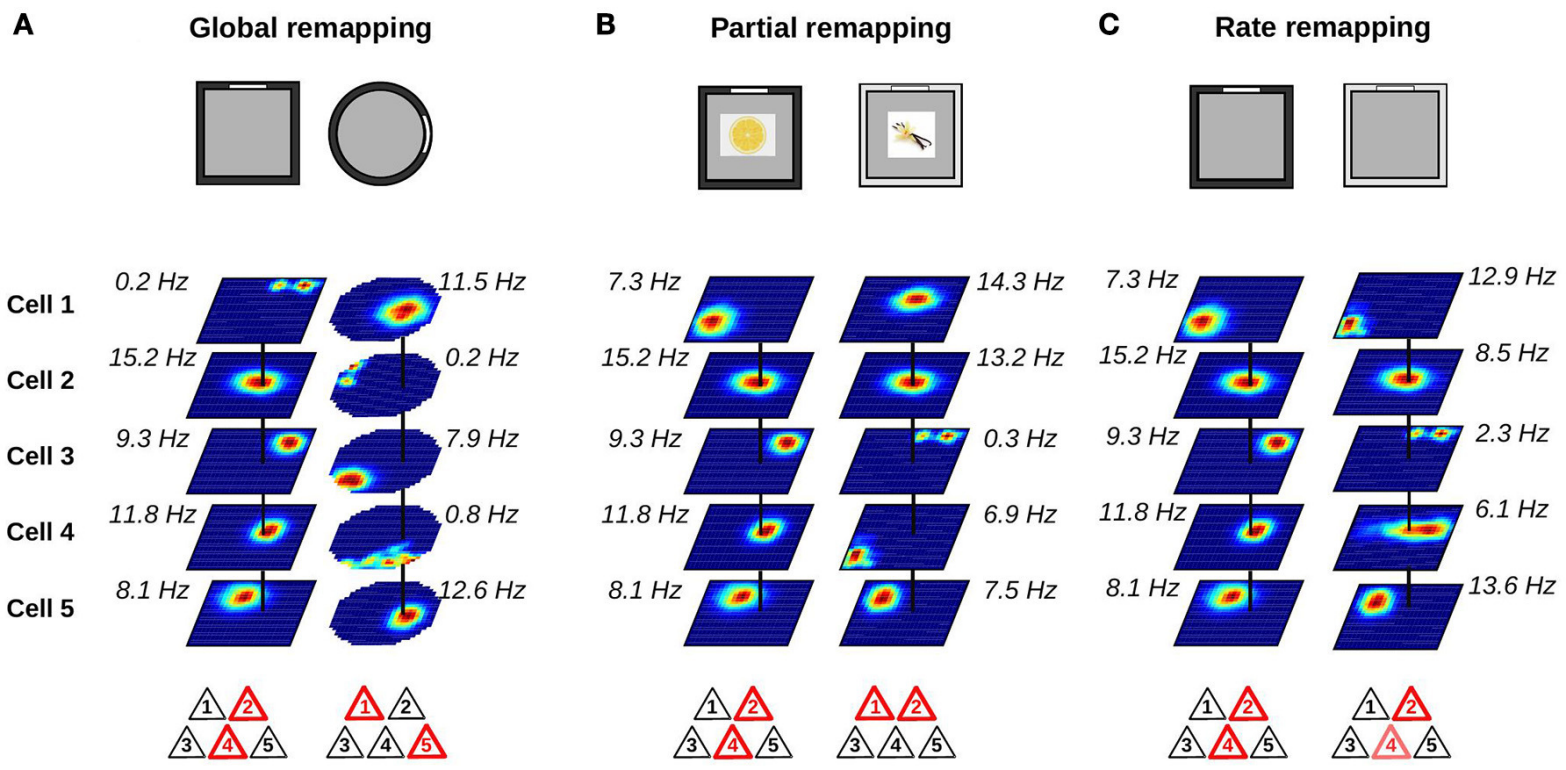
Changes in place cell ensembles associated with global, partial and rate remapping. **(A–C)** Top: recording environments. Middle: firing rate maps (dark blue: silent; red: maximum firing rate). Bottom: active cells are shown in red. **(A)** Global remapping. Place cells are recorded in environments of different shape (square or circular; Muller and Kubie, 1987). Place cells active in one environment are silent in the other environment, or active at a different location. The active ensemble is unique to the environment. **(B)** Partial remapping. Color (black or white) and odor (lemon or vanilla) of the environment are manipulated (Anderson and Jeffery, 2003). A subgroup of neurons change their spatial firing patterns depending on the features (color and odor) of the recording environment, while some neurons are unaffected by the manipulations. **(C)** Rate remapping. Recording environment varies in color (black or white; Leutgeb S. et al., 2005). The location of each firing field remains unchanged but the within-field firing rate varies. Place cells active together at a given location alter their firing rate.

The type of remapping associated with the largest changes in hippocampal activity is referred to as *global* or *complete* remapping (Figure 2A). When global remapping takes place, the activity patterns of hippocampal cells observed in two different environments are not correlated (Muller and Kubie, 1987; Leutgeb et al., 2004; Leutgeb S. et al., 2005; O'Neill et al., 2008). In these conditions, hippocampal cell ensembles are often said to be orthogonal, although we note that this does not necessarily equal to statistical independence. One of the first indications of global remapping was obtained by O'Keefe and Conway (1978). They recorded the activity of place cells on an elevated platform and on a T-maze. When they compared the activity of place cells in the two environments, they found that some place cells had firing fields in only one environment while others had fields in both. No relationship could be detected between the place fields of neurons with fields in both environments. This suggested that different internal maps were active in the two environments.

These initial observations were replicated using different recording environments (Muller and Kubie, 1987; Thompson and Best, 1989). For example, Muller and Kubie (1987) trained rats to forage for food pellets randomly scattered in a circular environment before performing a series of systematic manipulations of the recording environment. This foraging task, which is now used in several laboratories, ensured that the animal visited most locations within the recording environment and minimized any behavioral differences between different recording conditions. The manipulation that induced global remapping was to change the apparatus from a circular to a rectangular environment. They reported that the firing patterns of cells in one apparatus could not be predicted from their firing patterns in the other apparatus. Other less drastic manipulations, like for example removing a cue card on the wall of the environment, were not sufficient to cause this complete reorganization of the firing fields.

Kubie and Muller (1991) highlighted two important features of global remapping. First, out of the entire place cell population, only a small subset is active in one environment (Thompson and Best, 1989; Alme et al., 2014). If two environments are sufficiently different, the two subsets of place cells representing the environments are random samples from the place cell population. Second, if a cell is active in two environments, there is no relationship between its spatial firing pattern in the two environments (Muller and Kubie, 1987; Quirk et al., 1992; Alme et al., 2014). A consequence of these two features is that the cell ensemble active at a given location in one environment is unique to this environment. Thus, this active cell ensemble provides information about both the location within an environment and the identity of the environment.

### 2.2. Partial remapping: incomplete reorganization of cell ensembles

Global remapping is usually observed when comparing environments that differ in several features including shape, color, texture, and location within a laboratory. When only a fraction of the cues defining the local environment are changed, partial remapping is often observed (Figure 2B). The main distinction between global and partial remapping is that the place fields of a significant proportion of place cells remain unaltered during partial remapping. Thus, during partial remapping, the cell ensembles active in the two conditions are different but not orthogonal.

A clear demonstration of partial remapping was reported by Anderson and Jeffery (2003). In their experiment, rats foraged in a square environment and recordings were performed from the CA1 region of the hippocampus. The color (black or white) and odor (lemon or vanilla) of the environment were manipulated, leading to four possible contexts (black-lemon, black-vanilla, white-lemon, and white-vanilla). The firing maps of the cells were compared across the four conditions. A small group of neurons showed no remapping across conditions. A second minority of cells changed their firing fields to odor or color change only. One field, for example, was unaffected by a change in odor but turned silent when the color of the box was black. The majority of neurons, however, changed their firing fields in a complex way in response to color and odor manipulations. For example, a neuron had a firing field only in one of the four conditions. Another cell had a stable field in the two black contexts (black-lemon and black-vanilla), but the firing field was shifted in the white-lemon context and the neuron was silent in the white-vanilla context. Importantly, the changes of simultaneously recorded neurons were very heterogeneous. Whereas some neurons had stable firing fields between two conditions, other neurons switched on or off. These results clearly demonstrate that altering certain features of the recording environment can affect only a subgroup of neurons. An interesting aspect of partial remapping is that it is often triggered by changes that do not affect the geometric shape of the recording environment. These changes are referred to as non-metric or contextual changes.

### 2.3. Rate remapping: stable place fields with rate modulation

Lesion and neuropsychological studies indicate that the hippocampus is involved in declarative and episodic memory for a wide range of information, including non-spatial information (Eichenbaum, 2004; Squire et al., 2004). One strategy to demonstrate non-spatial coding in hippocampal neurons is to present different stimuli to the animal while controlling the position of the animal. In one experiment, rats were trained on an odor-guided non-matching-to-sample task (Wood et al., 1999). On each trial, a rat was presented with a cup containing sand scented with one of nine odors. The cup could be presented in one of nine positions. The rat had to dig in the cup only if the odor was different from the odor of the previous trial. In this task, a proportion of hippocampal neurons had different firing rates depending on what odor was presented to the rat, independently of the cup position. Similar findings were obtained in different tasks using a wide range of non-spatial stimuli (Komorowski et al., 2009; Allen et al., 2012; Aronov et al., 2017). But how can the spatial and non-spatial hippocampal codes be combined?

One possible mechanism was proposed by Leutgeb S. et al. (2005). They recorded the activity of hippocampal neurons in different enclosures and laboratory rooms. Two conditions were compared. In the variable enclosure-constant room condition, the recording took place in a single room but the recording enclosures varied in shape (circular or square) or color (black or white). In the variable room-constant enclosure condition, the same enclosure (black square) was used in two different laboratory rooms. The firing rate maps of CA3 and CA1 neurons were very similar across the two enclosures in the variable enclosure-constant room condition. However, close inspection of the firing rate maps showed that the firing rate of the cells within their otherwise stable firing fields varied. This was especially apparent in the CA3 region where cells showed large mean firing rate differences but no or small changes in the location of their firing fields. This type of rate changes was called *rate remapping* (Figure 2C). In the variable room-constant enclosure, neurons showed a change of both the location of their firing fields and their mean firing rates. In the CA3 region, the representations generated in the two laboratory rooms were statistically independent whereas those of the CA1 region still shared some degree of similarity. Thus, the variable room-constant enclosure triggered global remapping in the CA3 region.

This study is important because it shows that the hippocampus can simultaneously convey information related to the position of an animal and to the cues present in the environment. During rate remapping, the integrity of the spatial code is preserved because place fields are stable, but the precise firing rate of neurons varies to encode information not related to the current position of an animal. This coding scheme has been shown to take place not only during random foraging but also during memory tasks (Ferbinteanu et al., 2011; Ainge et al., 2012; Allen et al., 2012).

The concept of remapping originated from the idea that the hippocampus generates different spatial maps for different environments. In this respect, one might wonder whether a phenomenon in which place fields are stable should also be referred to as *remapping*. The use of rate remapping, however, is justified if one acknowledges that non-spatial variables are an integral part of hippocampal maps.

### 2.4. Distortion of familiar hippocampal spatial maps

Besides the three types of remapping described above, yet another type of hippocampal map modification has been observed when the aspect ratio of a familiar rectangular environment is suddenly altered. O'Keefe and Burgess (1996) were the first to report this phenomenon. Rats were initially trained in a rectangular box with constant dimensions. Then, during the recording days, the rats were exposed to a series of four rectangular environments with different lengths or widths. Comparing the firing fields of cells across the four conditions, they found that the fields often stretched or compressed along with the aspect ratio of the environment. The location of most fields appeared to remain at a fixed distance to one of the environment walls. These findings suggest that a familiar hippocampal map can be stretched or compressed when the animal is placed in a rectangular environment shortened or extended along one dimension. A subsequent study investigating the effect of a similar protocol on the activity of spatially selective neurons of the medial entorhinal cortex suggests that this distortion of hippocampal maps might only occur when the manipulated environment is novel to the animal (Barry et al., 2007).

In another study, Fenton and colleagues showed that hippocampal spatial maps can be distorted when the distance between two salient proximal cues is manipulated (Fenton et al., 2000). The authors recorded the activity of place cells in rats foraging in a gray cylinder with a black and a white cue card attached to the wall. One manipulation involved varying the angle separating the two cue cards. When the two cues were moved closer together, the firing fields located near the cues also moved closer together. These findings provide additional support to the idea that familiar hippocampal maps are not rigid but can undergo some degree of deformation. These map deformations appear to occur mainly following subtle manipulations of the recording environment which are not sufficient to induce partial or global remapping.

### 2.5. Different orthogonalization of cell ensembles across hippocampal subfields

The hippocampus comprises four different subfields (dentate gyrus, CA3, CA2 and CA1). These subfields have very different anatomical organizations. One frequently cited difference is that excitatory connectivity between pyramidal neurons is more prominent in the CA3 than in the CA1 region (Lorente de Nò, 1934; Li et al., 1994). The two regions also receive different inputs from the entorhinal cortex (Amaral and Witter, 1995). These differences in network architecture could be reflected in the remapping properties of the different subfields. This possibility was tested by Leutgeb et al. (2004). They recorded the activity of pyramidal cells in the CA3 and CA1 regions in rats chasing food pellets in square boxes located in different laboratory rooms. They found that neurons in the CA3 region were more likely to be silent than neurons in the CA1 region. When comparing cell ensembles observed in two different rooms, they showed that CA3 cell ensembles underwent global remapping. In contrast, CA1 cell ensembles showed a significant degree of overlap. One factor contributing to the similarity of CA1 cell ensembles in the two rooms was the similarity in shape of the recording boxes. When the shape of the enclosures in the two rooms was sufficiently different (large square vs. small circle), the CA1 cell ensembles observed in the two rooms were also independent. These results suggest that when the recording conditions share some degree of similarity, the CA3 region generates more dissimilar representations than the CA1 region.

However, the distinction between CA3 and CA1 cell ensembles appears to depend on the precise recording conditions. In a different study, CA3 and CA1 place cells were recorded as rats ran clockwise on a circular maze (Lee et al., 2004). The recording environment was surrounded by a black curtain. Two types of landmarks were present within the curtain: proximal and distal cues. The proximal cues were four textured surfaces on the maze while distal cues comprised landmarks attached to the curtain as well as standing objects (e.g., a white box). Rats were first trained with a standard configuration of cues over several days. The recording sessions included standard trials and also cue-mismatch trials. In the cue-mismatch trials, the proximal and distal cues were rotated by the same amount but in opposite directions. CA3 place fields were more likely to rotate coherently than CA1 place fields in the cue-mismatch trials. In the CA3 region, most fields that rotated did so in the direction of the proximal cues. In the CA1 region, the cells that altered their firing were more likely to show changes that could not be explained in terms of simple rotation with proximal or distal cues. A larger proportion of CA1 cells had a robust firing field in only one of the two trial types. Thus, when comparing two conditions in which the same cues are present in different configurations, cell ensembles in the CA1 regions show a higher degree of reorganization than those in the CA3 region.

### 2.6. Orthogonalization dynamics of hippocampal cell ensembles

The type of remapping observed between two environments is not always stable over time. Instead, in some conditions, hippocampal remapping is a dynamic process shaped by experience. This was shown by Bostock et al. (1991). They recorded the activity of hippocampal cells as rats ran in a gray cylinder with a prominent cue card covering 100° of the internal wall of the environment. In this experiment, the cue card acted as a dominant polarizing cue; a rotation of the cue card along the wall of the cylinder caused a similar rotation of hippocampal place fields (Muller et al., 1987; Bostock et al., 1991). The key manipulation in the study was to change the white cue card for a black one within each recording session, and the firing rate maps between the two conditions were compared. Cells for which the two firing maps could be matched by a rotation were said to exhibit *rotational* remapping, whereas cells whose maps could not be matched by a rotation exhibited what was called *complex* remapping. Sixteen out of 36 cells exhibited rotational remapping and the remaining 19 showed complex remapping. The similarity of the maps for rotational cells was high and the angular position of the firing fields relative to the two cue cards was constant. In contrast, complex cells showed more pronounced changes in their firing fields. Importantly, the ratio of rotational to complex cells changed with experience. On the first exposure to the black cue card, remapping was mainly rotational. On subsequent days, more complex remapping was observed. Once the transition from rotational to complex remapping occurred in one animal, no more rotational remapping was observed. These findings have several implications. First, the delay between the presentation of the black cue card and the onset of complex remapping rules out the possibility that remapping is a simple response to change in sensory cues. Instead, the type of remapping that is observed can change with experience. Second, once two environments are represented by independent maps, the orthogonalization process appears to be irreversible.

Similar findings were also observed when comparing the activity of CA1 place cells in square and circular environments (Lever et al., 2002). On the very first exposure to the two environments, several cells had firing fields at the same location in the two environments. With more training over several days, the firing patterns observed in the two environments became progressively more divergent. Examination of the firing activity of single cells indicated that the divergence process took place over several days. For example, a cell had the same firing field in the two environments during the first 19 recording days. Then, a new subfield emerged in one environment and its firing rate progressively increased while the firing rate of the old field decreased. The transition process of this neuron took place over 2 days. Again, the orthogonalization process appeared irreversible: most cells showed remapping between the two environments even after more than a month without exposure to either environments.

### 2.7. Transition dynamics between pre-established hippocampal maps

Once two environments activate two distinct hippocampal representations, these two representations appear to behave like discrete attractor states (Wills et al., 2005). This was shown by repeatedly exposing rats to a circular and a square environment until the two elicited orthogonal CA1 representations. The square and circle initially differed in color, texture and shape. After 3 days, the two environments were constructed from a morph-box that was configured as a square or circle. At this stage, most rats (4 out of 6) showed what appeared to be global remapping between the square and circle. The rats exhibiting remapping were then presented with a series of environments whose shapes were different intermediates between a circle and a square. The hippocampal representation for intermediate shapes was either that of the square or the circle, with little evidence for intermediate representations. The transition between the square and circle representations was abrupt and occurred at the same moment for simultaneously recorded place cells. Thus, as the environment was varied along a continuum between two pre-established shapes, the hippocampal network showed abrupt and coherent transitions between the two representations, suggesting the presence of discrete attractor states in the hippocampus.

A similar set of experiments was performed by Leutgeb J. K. et al. (2005), and they reached surprisingly different conclusions. Here, the rats were trained for 16–19 days in the same morph-box configured as a square or a circle. This protocol led to rate remapping between the square and circle; the firing rates changed substantially but the location of most firing fields remained constant. After this discrimination stage, the environment was transformed from one shape to the other through a series of five intermediate shapes. The rate changes observed during the gradual transformation took place at different times in the morph sequence for different cells. Thus, in this study, the gradual transformation of the environment did not lead to an abrupt transition but to a gradual change of cell ensembles.

At least three differences can explain the different findings in these two “morph-box” studies. First, different types of remapping were observed in the two studies (global vs. rate remapping). Second, during training trials, the square alternated with the circle in one study (Wills et al., 2005) whereas the sequence of presentation was random in the other (Leutgeb J. K. et al., 2005). Finally, the intermediate shapes were presented in a random order in Wills and colleagues' study. In Leutgeb and colleagues' work, the transitions between the square and circle occurred in incremental steps^1^. Which difference or combination of differences led to the different remapping dynamics in the morph sequence has yet to be established. Nevertheless, these two studies highlight the rich repertoire of remapping dynamics in the hippocampus. Depending on the conditions, the hippocampal network may coherently and abruptly switch between two pre-establised representations or, alternatively, progressively drift from one representation to the other. An important next step will be to establish the precise conditions leading to these two remapping dynamics.

### 2.8. Remapping variability between experimental subjects and studies

It is now well established that at least three types of remapping can be observed in the hippocampus: global, partial and rate remapping. What is still unclear is the exact conditions under which the three types of remapping are seen. Indeed, a careful review of the literature reveals some inconsistencies between studies. For example, Muller and Kubie (1987) compared the activity of place cells in circular and rectangular environments and found that most place cells turned on and off between the two conditions or had place fields at unpredictable locations. This protocol is very similar to that of Leutgeb et al. (2004) in which they compared the activity of CA1 and CA3 neurons in square and circular environments, both located in the same recording room. In this latter study, the firing rate map similarity between the two conditions was high and the firing fields showed only minor shifts. Thus, similar manipulations triggered global remapping in one study and rate remapping in the other. Another discrepancy is found when investigating the effect of changing the color of the recording environment. In one study, this caused partial remapping (Anderson and Jeffery, 2003) whereas it led to rate remapping in another (Leutgeb S. et al., 2005).

Perhaps even more surprising is the observation that the type of remapping observed in a given experiment often varies between animals. For example, in the study performed by Wills et al. (2005) in which a morph-box was used to characterize the transition between pre-established representations, only 4 out of 6 rats showed strong remapping between the circle and square environments. The reasons behind this type of inter-subject variability are still unknown.

Thus, future studies will need to provide a precise description of the determinants behind the distinct forms of remapping. Part of this remapping variability might be due to subtle differences in training protocols, to uncontrolled stimuli in or around the recording environments, or to differences in the salience of the manipulated cues for the animal. An additional factor adding complexity is the orthogonalization process that changes the remapping properties over time (Bostock et al., 1991; Lever et al., 2002). Nevertheless, identifying the key determinants of the different remapping types will provide new insights into how the hippocampus generates and stores internal representations.

Another contentious issue concerns the stability of hippocampal place fields over periods of several days. Several studies using tetrode recordings have reported that the firing fields of place cells can be stable across recording days (Muller et al., 1987; Thompson and Best, 1990; Kentros et al., 1998; Cacucci et al., 2007; Mankin et al., 2012). For example, Thompson and Best (1990) recorded the activity of 10 CA1 place cells over 6 or more days in rats running on a radial arm maze. They found that all cells had a stable firing field. However, a more recent study using Ca^2+^-imaging in freely moving mice questioned the assumption that most place fields are stable across days (Ziv et al., 2013). Ziv and coworkers imaged the Ca^2+^ activity of CA1 neurons expressing the Ca^2+^-indicator GCaMP3 as mice ran on a linear track. With this technique, they were able to track the activity of hundreds of cells over several days. They found that on each session, approximately 30% of the recorded neurons were active. Surprisingly, only 15–25% of this subset of cells with a place field on one day still had a field on a different day. When cells were active on several days, they usually had very similar firing fields. This study suggests that there might be much more variation in the composition of hippocampal cell ensembles across days, even when the recording environment remains unchanged. More studies, ideally employing a wide range of recording techniques and behavioral protocols, are needed to firmly establish the degree of long-term stability of hippocampal place cells.

## 3. The entorhinal cortex as a trigger of hippocampal remapping

**Figure 3:**
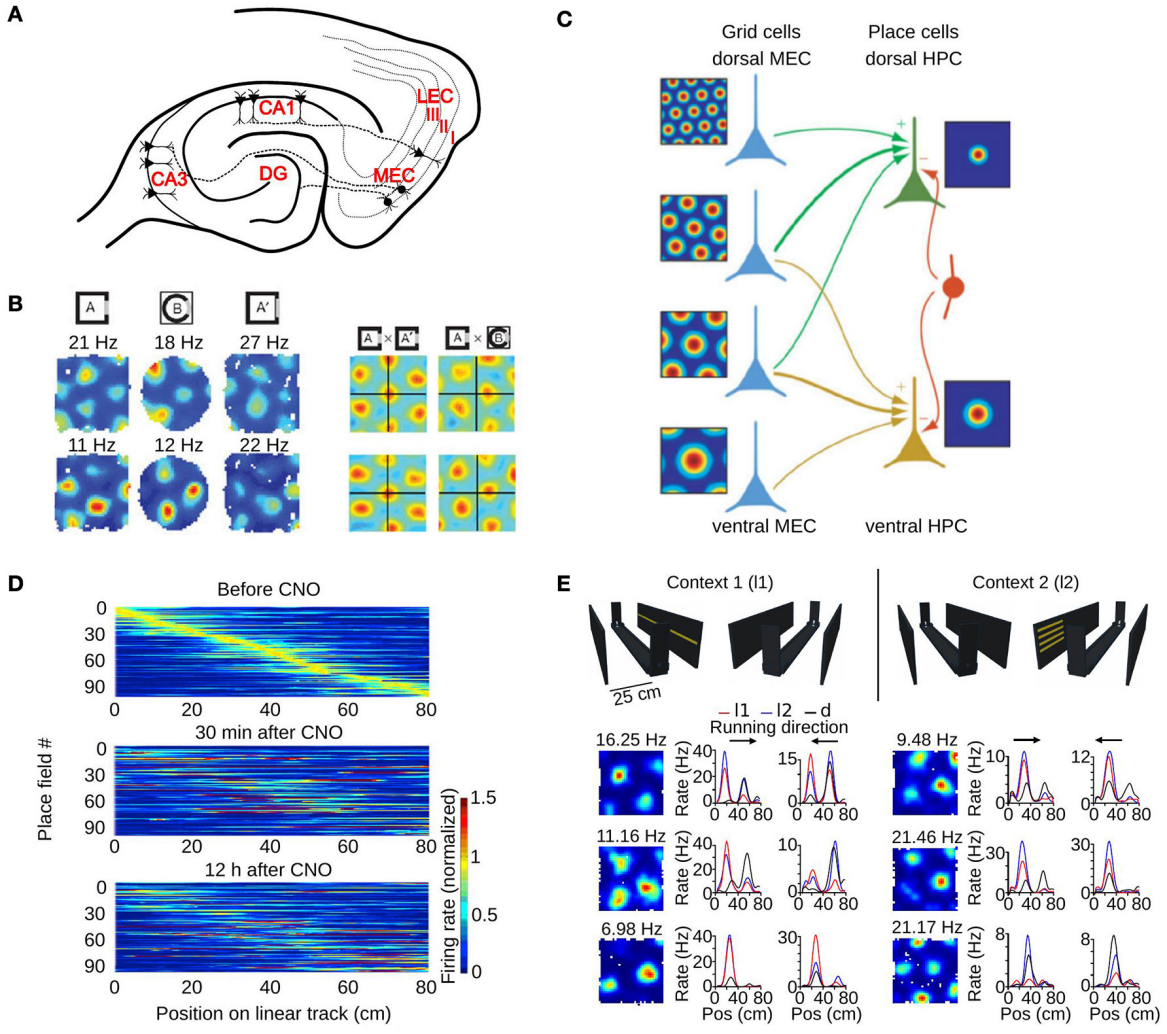
The role of the medial entorhinal cortex in hippocampal remapping. **(A)** Schematic of a horizontal section through the hippocampal formation showing the main projections from the MEC to different hippocampal subfields. DG, dentate gyrus; CA, cornus ammonis; MEC, medial entorhinal cortex; LEC, lateral entorhinal cortex. Roman numbers indicate the superficial layers of the entorhinal cortex. **(B)** Realignment of grid cells in the MEC during global remapping in the hippocampus. Left: color coded rate maps of two simultaneously recorded grid cells (row 1 and 2) during three recording trials (peak firing rates are indicated above each map). Right: spatial cross-correlations between pairs of rate maps on the left, for exposures to the same (AxA') or different environments (AxB). Note the shift of the central peak in the latter condition. Adapted from Fyhn et al. (2007). **(C)** Model of place cell formation from grid cell input. Grid cells (blue) with different spatial scales and orientations but similar spatial phase (common central peak) project to a place cell. A single place field is generated from weighted summation of the inputs and global inhibition (indicated by the red interneuron). HPC, hippocampus. Adapted from Solstad et al. (2006). **(D)** Hippocampal remapping induced by partial inactivation of the MEC using chemogenetic tools. Place cells in CA3 were recorded while mice were running along a linear track before, 30 min after, and 12 h after inactivation. Color coded rate maps show the activity of the recorded place cells along the linear track ordered according to their firing location in the baseline session (top panel). Adapted from Miao et al. (2015). **(E)** Non-metric contextual visual cues affect firing rates of MEC neurons in a 1D environment. Top: schematic of the linear track with two adjacent walls on which LED arrays were attached. Mice ran along the linear track while contextual visual cues changed. In context 1 (l1, left), a single row of LEDs on one wall was turned on. In context 2 (l2, right), four short LED stripes on the opposite wall were lit. During dark trials all LED were switched off. Bottom: firing rate maps of six grid cells recorded in a square environment (1st column), and 1D rate maps of the same cells recorded on the linear track (2nd and 3rd column, each running direction plotted separately). Note the firing rate changes of grid cells between the different contextual cues (l1-red, l2-blue). Pos, position. Adapted from Pérez-Escobar et al. (2016).

The principal cortical input to the hippocampus originates in the superficial layers of the entorhinal cortex (Amaral and Witter, 1995). The entorhinal cortex has two subdivisions, the medial entorhinal cortex (MEC) and the lateral entorhinal cortex (LEC), and both send axons to the dentate gyrus, CA3 and CA1 subfields of the hippocampus (Figure 3A). In return, the CA1 subfield projects back to the deep layers of the entorhinal cortex either directly or indirectly via the subiculum. Based on this connectivity, it is very likely that some of the spatial properties of hippocampal place cells are determined by the activity of neurons in the entorhinal cortex.

The MEC contains several types of spatially selective neurons (Hafting et al., 2005; Sargolini et al., 2006; Savelli et al., 2008; Solstad et al., 2008). The best studied are the grid cells that have several firing fields organized as a grid of equilateral triangles (Hafting et al., 2005). The spatial firing pattern of a grid cell can be characterized by three main variables: phase, orientation and spacing. Grid cells are organized in functional modules (Stensola et al., 2012). Each module is located at a different dorso-ventral level within the MEC, with a possible small anatomical overlap between modules. Within a module, grid cells share the same grid orientation and spacing but differ in phase. Between modules, grid cells have different spacing and can vary in orientation (Barry et al., 2007; Stensola et al., 2012).

### 3.1. Grid cell activity during global remapping

The first study investigating the role of grid cells in hippocampal remapping was performed by Fyhn et al. (2007). They recorded from the hippocampus and the MEC in conditions that triggered either rate or global remapping in the hippocampus (Leutgeb S. et al., 2005). They found that the type of remapping observed in the hippocampus could be predicted by the activity of grid cells. During hippocampal rate remapping triggered by changes in color or shape of an environment, no major change in the activity of grid cells was reported. During hippocampal global remapping induced by recording in two different laboratory rooms, a change in phase and orientation of grid cells was observed (Figure 3B).

Two fundamental differences between place cells and grid cells were uncovered by early grid cell experiments (Hafting et al., 2005; Fyhn et al., 2007). In contrast to hippocampal place cells which change their firing associations during global remapping, grid cells do not display such reorganization. Instead, the same geometric transformation appears to be applied to all locally recorded grid cells and their firing associations remain unchanged. In addition, whereas only a subset of place cells is active in one environment, grid cells are active in all environments sufficiently large to contain at least one firing field. These differences highlight the distinctive ability of the hippocampal network to generate orthogonal cell ensembles for different environments.

### 3.2. Grid-place transformational models explaining global remapping

To understand how changes in the activity of grid cells can lead to remapping of place cells, one must first consider how place fields could be generated from the activity of grid cells. Soon after the discovery of grid cells, formal models of place field generation were put forward. They proposed that place fields are generated from the linear summation of inputs from a subset of grid cells (Figure 3C) (O'Keefe and Burgess, 2005; Solstad et al., 2006). Place cells with single firing fields could emerge from the inputs of a few (< 50) grid cells with different grid orientation and spacing but similar grid phase (Solstad et al., 2006). A place field emerged because of the alignment of grid cell fields centered on the place field. When there is more grid phase variability in the input to a place cell, no dominant firing field was observed.

A key aspect of grid-place transformational models is the connectivity between grid and place cells that allows the generation of non-periodic place fields from grid cell inputs. Computational studies therefore tested whether such connectivity could emerge in a competitive network with modifiable connections (Rolls et al., 2006; Si and Treves, 2009). One model comprised two layers, one for grid cells and one for dentate gyrus place cells (Rolls et al., 2006). Each grid cell could have one of 10 different spacings and different phases. Their maximum firing rate varied from field to field. The connectivity between grid and place cells was first set randomly. This feedforward connectivity was then altered via a Hebbian learning rule in which the connections between co-active neurons were strengthened. Within the dentate gyrus layer, mutual inhibition was implemented to ensure that only a small fraction of dentate gyrus neurons was active. After a brief training period in which the connectivity was modified, cells of the dentate gyrus typically had a single place field. This model shows that simple competitive network learning can generate place fields from the periodic activity of grid cells. This model has also been modified to account for the multiple non-periodic fields of dentate gyrus cells by adding a non-spatial input from the LEC to the dentate gyrus (Si and Treves, 2009). It should be noted that modifiable connections are not required to generate place cells from grid cell inputs. Indeed, de Almeida and coworkers showed that a grid-place model including the summation of inputs from randomly selected grid cells, synapse strength variability and a winner-take-all rule between granule cells can generate place cells with only few firing fields (de Almeida et al., 2009a).

One finding that was not fully taken into account by these first models is that grid cells have a modular organization (Barry et al., 2007; Stensola et al., 2012). Different grid modules were shown to respond independently to change in an environment. For example, when recorded in a compression protocol inducing rescaling of the grid pattern (Barry et al., 2007), grid cells from different modules reacted independently (Stensola et al., 2012). Grid cells in one module showed very little compression whereas those of other modules rescaled completely.

More recent theoretical work aimed at illustrating how place cell properties could originate from the activity of a small number of grid cell modules. Monaco et al. (2011) proposed a model in which place cells received inputs from random sets of grid cells and in which global recurrent inhibition between place cells caused network competition. The model generated place fields with properties comparable to experimental data. Grid cells were grouped into a small number of modules and each module reacted coherently to changes in the recording environment. The model showed that independent realignment of few grid cell modules caused global remapping in the place cell population. One difference between this model and more recent experimental observations is that, in the model, grid cells have variable spacings within a module and all modules share the same grid orientation. Nevertheless, this model provides valuable insights regarding how global hippocampal remapping could be triggered by the independent realignment of grid cell modules.

### 3.3. Contextual-gating of grid cell input as a mechanism for partial hippocampal remapping

The grid-place transformational models provide a simple way to explain global remapping. However, many fall short to provide an explanation for partial remapping in which only some hippocampal place cells change their firing fields. Hayman and Jeffery were the first to put forward a model that can account for partial remapping (Hayman and Jeffery, 2008). The main concept in this model is that the grid cell input from the MEC to place cells is modulated by a second input from the LEC. The LEC input carries what is called contextual information. This modulatory or gating mechanism is envisaged to take place in the dendritic tree of granule cells of the dentate gyrus. The inputs from the MEC and LEC target the distal and middle third of the dendritic trees, respectively. Hayman and Jeffery proposed that, because of Hebbian plasticity, grid cells with overlapping firing fields in a particular region of the environment will tend to converge onto a particular region of the dendritic tree. Via a similar mechanism, correlated contextual inputs will also tend to target specific regions of the dendritic tree. The result is that the dendritic branch receiving a spatial input that is active at a given location also receives an input about a given context. Both the spatial and contextual inputs need to be active to drive the neuron to fire. Thus, the contextual input acts as a gate that modulates the influence of spatial inputs on the firing of hippocampal neurons. Changes in context allow different dendritic branches to drive granule cell activity. When only a subset of the branches are altered by contextual changes, only some cells or some firing fields will remap, just like what had been observed during partial remapping experiments. This model also provides a mechanism by which hippocampal partial or rate remapping can occur in conditions in which grid cell activity is unchanged.

A similar approach was used by Rennó-Costa and colleagues to explain rate remapping in dentate gyrus cells (Rennó-Costa et al., 2010). Their model consisted of three populations of cells: grid cells of the MEC, broadly spatially tuned cells of the LEC which reacted to context changes, and dentate gyrus neurons. Dentate gyrus neurons received inputs from a random subset of MEC and LEC neurons. These inputs were summed by dentate gyrus neurons. The firing rates of dentate gyrus neurons were subjected to a winner-take-it-all process which set the level of inhibition so that only cells with the strongest inputs were allowed to be active (de Almeida et al., 2009b). Changes in context (i.e., changes in the shape of the recording box) were modeled as a change in the location of the broad firing fields of LEC neurons, while the spatial inputs from grid cells remained unchanged. The change of LEC inputs, together with the network competition between dentate gyrus cells led to rate remapping in dentate gyrus neurons. Interestingly, individual firing fields of dentate gyrus cells showed independent rate changes, similar to what had been observed *in vivo* (Leutgeb et al., 2007).

One assumption of these models is that spatial and contextual information reaches the hippocampus via separate pathways. This idea was tested by Marozzi et al. (2015). They recorded the activity of grid cells in rats exploring a square box in which the color and odor could be manipulated (black or white; lemon or vanilla). This protocol had been shown to elicit partial remapping in the hippocampus (Anderson and Jeffery, 2003). They found that the firing rate of grid cells did not change across the different contexts. However, grid cells reacted by changing the location of their firing fields (phase change). Phase changes were larger following odor change than color change, and like in previous studies, all grid cells appeared to be subjected to the same translation. No change in grid orientation was observed. These findings indicate that grid cells receive non-metric, or contextual, as well as spatial information. Thus, spatial and contextual information is already combined at the level of the MEC.

During partial hippocampal remapping, a significant proportion of place cells have stable firing fields across conditions. It is still not known why these firing fields are stable in conditions where grid cells realign (Marozzi et al., 2015). One possibility is that the realignment in grid cells only affects a subset of grid modules and that hippocampal cells with stable fields receive inputs preferentially from stable grid cell modules. Another possibility is that the spatial activity of hippocampal place cells is not entirely controlled by the activity of grid cells of the MEC. This is supported by the observation that large excitotoxic lesions of the MEC do not abolish spatial selectivity in hippocampal place cells (Hales et al., 2014). In addition, some place cells might receive spatial inputs from border cells located in the MEC or elsewhere (O'Keefe and Burgess, 1996; Lever et al., 2009; Boccara et al., 2010). If the firing field location of border cells is not affected by contextual manipulations, they could provide an anchor for the hippocampal place fields that remain unchanged during partial remapping.

### 3.4. Causal evidence for a role of the medial entorhinal cortex in hippocampal remapping

The studies presented so far suggest that the MEC and grid cells are important for hippocampal remapping. However, they are based on correlative evidence. Fortunately, with the development of new techniques to manipulate the activity of neurons at a high spatial and temporal resolution, it is now possible to test whether changes in the activity of MEC neurons can trigger remapping in the hippocampus. This idea was tested independently in two laboratories (Miao et al., 2015; Rueckemann et al., 2016). In one study, the outward-directed proton pump ArchaerhodopsinT (ArchT) was used to hyperpolarize MEC neurons of rats upon light stimulation (Rueckemann et al., 2016). Simultaneous recordings were performed in the CA1 region. The rats were trained to run in one direction on an elliptical track. It was found that partial inactivation of the MEC led to a partial remapping of hippocampal cells. Partial remapping was inferred from a small reduction in the spatial stability of place cells upon light stimulation.

The second study investigated the consequences of partial inhibition of the MEC in mice (Miao et al., 2015). In one experiment, MEC neurons were inhibited by expressing the designer muscarinic receptor hM4D in MEC neurons (Armbruster et al., 2007). Activation of the receptor with the ligand clozapine-N-oxide (CNO) hyperpolarized MEC neurons and decreased their firing rate to less than 50% of their normal rate. The effect of this partial inactivation of the MEC on CA3 neurons was assessed in mice running on a linear track. Comparing the firing rate maps of a baseline period to those during MEC inactivation revealed significant changes (Figure 3D). The stability of maps from before to after MEC inactivation was higher than chance, suggesting that the hippocampal cell ensembles that were active before and after MEC inactivation shared some similarity. Importantly, MEC inactivation did not cause significant changes in the mean firing rate or spatial selectivity of hippocampal place cells, ruling out the possibility that the lower stability of hippocampal fields was due to a loss of spatial selectivity. Taken together, these two studies indicate that the activity of MEC neurons control the location of some hippocampal place fields. Inhibiting MEC neuron activity alters hippocampal cell ensembles representing an environment.

A follow up study used transgenic mice to investigate whether manipulating the firing rate of layer II MEC neurons is sufficient to cause hippocampal remapping (Kanter et al., 2017). Two mouse lines were created in which one of the designer receptors hM4D or hM3D was expressed most strongly in layer II stellate cells, with additional but more limited expression in the pre- and parasubiculum. Approximately 25% percent of layer II stellate cells expressed the designer receptor. Excitation of MEC layer II neurons by injections of CNO in hM3D-expressing mice led to considerable changes in the firing fields of CA1 neurons. 27% of the cells shifted the location of their place fields, whereas 14% turned their firing fields on or off. Another 15% of the neurons changed their within-field firing rate or field size without changing the location of their primary firing fields. CA1 neurons also increased their mean firing rate and place field size, and showed a decrease in spatial information content. Interestingly, this artificial hippocampal remapping was not observed after inhibition of MEC layer II neurons in the mouse line expressing hM4D. When assessing the effect of CNO injections in mice expressing hM3D on the activity of MEC neurons, putative excitatory neurons were found to have increased firing rate and field size. The locations of the firing fields of MEC neurons, however, were unchanged. These results demonstrate that induced firing rate changes in a subset of layer II MEC neurons can trigger partial remapping in the hippocampus.

### 3.5. Rate changes in grid cells and other neurons of the medial entorhinal cortex

What is the evidence that grid cells actually change their firing rate when the environment explored by an animal is modified? One line of evidence came from recording the activity of grid cells while visual landmarks surrounding a linear track were manipulated (Pérez-Escobar et al., 2016). In this experiment, the track was flanked by two walls and each wall had a distinct light pattern that could be switched on and off (Figure 3E). Mice ran back and forth on the track and the light pattern changed every five runs. The firing rate of grid cells was compared for runs with either of the visual landmarks. The firing rate of approximately 40% of grid cells changed between the two conditions (Figure 3E). The rate maps on the maze in the two conditions were highly correlated, suggesting that the main change in grid cell activity was a firing rate change instead of a change in field location. Similar firing rate changes were also observed for other spatially selective neurons of the MEC. These results show that the firing rate of grid cells can change to reflect the visual landmarks perceived by the animal.

A second set of data indicates that rate changes in grid cells might contribute to hippocampal rate remapping (Diehl et al., 2017). The activity of grid cells was recorded in boxes with different shapes (circle or square) or colors (black or white), a paradigm causing rate remapping in the hippocampus (Leutgeb S. et al., 2005; Diehl et al., 2017). During the manipulations, the location of the boxes within the experimental room remained unchanged. In those conditions, the spatial firing pattern of grid cells remained stable across manipulations of box shape or color in most rats. However, the within-field firing rate of grid cells changed more than predicted by chance. This indicates that grid cells redistribute their firing rate across their firing fields to encode the features of the recording enclosure. In line with this conclusion, a re-analysis of the dataset of Marozzi et al. (2015), in which grid cells were recorded in square boxes with different wall colors or odors, showed that grid cells indeed change their within-field firing rates in response to alteration in non-metric cues (Ismakov et al., 2017). These firing rate changes in grid cells upon landmark manipulations, together with the hippocampal remapping caused by direct manipulation of MEC neuron activity, provide compelling evidence for an important role of grid cells and other MEC neurons in triggering hippocampal remapping.

### 3.6. Hippocampal spatial maps without grid periodicity

Despite accumulating evidence that MEC neurons contribute to place cell spatial selectivity and remapping, the specific role of grid periodicity in these processes is still not fully understood. The relationship between grid cell periodicity and place cell firing has been studied through the normal development of grid and place cells. Electrophysiological recordings in rats during the first month after birth show that place cell firing emerges at postnatal day 16 whereas grid cell periodicity is only observed from postnatal day 20 (Langston et al., 2010; Wills et al., 2010). This finding suggests that grid cell periodicity is not required for place cell spatial selectivity at this age. Between postnatal days 16 and 20, place cell representations are denser, more stable and more accurate near the walls of the recording environment (Muessig et al., 2015). After postnatal day 20, place cell representations become more homogeneous throughout the environment. Since this change coincides with the emergence of grid cells, it is possible that the periodic activity of grid cells contributes to the stability of hippocampal place fields located far away from the environment borders (Muessig et al., 2015). Interestingly, global remapping was observed from postnatal day 16 in rats (Muessig et al., 2016), demonstrating that grid periodicity is not required for global remapping.

The necessity of grid periodicity for the spatial selectivity of place cells has also been questioned based on experiments in which MEC activity was manipulated via medial septum inactivation (Brandon et al., 2011; Koenig et al., 2011). Medial septum inactivation abolishes grid periodicity and it reduces the spatial selectivity of non-periodic MEC cells. In the hippocampus, the firing rate of place cells is decreased but their spatial selectivity is only marginally affected (Koenig et al., 2011; Brandon et al., 2014), again suggesting that place cell selectivity does not require grid periodicity. Importantly, global remapping is also observed during septal inactivation (Brandon et al., 2014).

Based on these results from two different experimental approaches, it seems very likely that a periodic and stable grid cell input is not essential for the formation of stable spatial representations in the hippocampus and for global remapping. One possibility is that, in absence of intact inputs from grid cells, normal place fields can be generated from the activity of other spatially selective MEC neurons (e.g., border cells). It was recently shown that non-periodic neurons of the MEC display large changes in their firing fields and firing rates in response to modifications of the recording environments (Pérez-Escobar et al., 2016; Diehl et al., 2017). In future studies, the role of these neurons for place cell selectivity and remapping will need to be explored further.

### 3.7. Lateral entorhinal cortex and hippocampal remapping

Much less is known about the role of the LEC in place cell activity and remapping. In contrast to the MEC that is populated by grid cells, border cells and head-direction cells, LEC neurons usually express much less spatial selectivity (Hargreaves et al., 2005; Yoganarasimha et al., 2011). Some LEC neurons however have been shown to fire at object locations (Deshmukh and Knierim, 2011). For this reason, a commonly held view is that the LEC might provide non-spatial information to the hippocampus (Knierim et al., 2006; Manns and Eichenbaum, 2006; Hayman and Jeffery, 2008).

The potential role of the LEC for hippocampal remapping has been explored in one study (Lu et al., 2013). Rats with NMDA lesions of the LEC and control rats were trained to run in the square and circular configuration of a morph-box. The protocol induced strong rate remapping in the CA3 region of control rats. In LEC-lesioned rats, the magnitude of rate remapping was reduced. Similar results were obtained when rate remapping was induced by recording in two square boxes of different colors (black or white). These results suggest that the LEC is required for normal rate remapping. Lu and coworkers also performed recordings from the LEC to investigate whether rate remapping is present in the LEC. In contrast to CA3 neurons, LEC neurons did not exhibit substantial firing rate differences between recording trials in boxes of different colors. Thus, although the LEC is required for normal hippocampal rate remapping, it does not appear likely that hippocampal rate remapping is simply inherited from the activity of LEC neurons. In contrast, a large proportion of MEC neurons show strong spatial and rate changes in conditions triggering rate remapping in the hippocampus (Diehl et al., 2017).

## 4. Conclusion

The world surrounding us is subject to frequent changes. The hippocampus reacts to these changes via rate, partial or global remapping, which are associated with a progressive increase in the orthogonalization of hippocampal cell ensembles. The exact determinants of each type of remapping are still not fully understood. A general principle is that partial geometric or contextual manipulations of the environment tend to induce rate or partial remapping while more prominent changes involving several sensory modalities are more likely to cause global remapping (Kubie and Muller, 1991). Importantly, several studies have demonstrated that the relationship between remapping and sensory inputs is complex. Part of this complexity comes from the modulation of hippocampal remapping by factors such as the previous history of the animal with the recording environments (Bostock et al., 1991; Lever et al., 2002). An important next step in understanding how the hippocampus works will be to characterize the effect of these modulators on remapping. For example, one unanswered question is: what are the changes in the hippocampus or the entorhinal cortex that mediate the effect of previous experience on remapping? Understanding this process will shed light on how previously acquired memories influence our internal representations of space.

## Author contributions

PL, OK, LK, and KA wrote the manuscript. OK and PL prepared the figures.

### Conflict of interest statement

The authors declare that the research was conducted in the absence of any commercial or financial relationships that could be construed as a potential conflict of interest.

## Footnotes

^1^see Leutgeb S. et al. (2005) for a more in-depth discussion of the differences
